# Urine transferrin as an early endothelial dysfunction marker in type 2 diabetic patients without nephropathy: a case control study

**DOI:** 10.1186/s13098-021-00745-1

**Published:** 2021-11-07

**Authors:** José Juan Sánchez-Hidalgo, Juan Antonio Suárez-Cuenca, José Juan Lozano-Nuevo, Víctor Hugo García-López, María Graciela Leal-Gutiérrez, Sein Antonio León-Angel, María Leslye Ramírez-Villa, Martha Elena Rodea-Rubio, José Enrique González-Hernández, José Antonio Canela-Mayoral, Eduardo Murillo-Heredia, Eduardo Vera-Gómez, Alejandro Hernández-Patricio, Carlos Ramiro Zamora-Alemán, Gabriela Alexandra Domínguez-Pérez, Juan Ariel Gutiérrez-Buendia, Paul Mondragón-Terán

**Affiliations:** 1 Internal Medicine Department, Ticomán General Hospital, Mexico City Health Department. 7, Plan de San Luis, La Purísima Ticomán, Alcaldía Gustavo A. Madero, P.O. 07330, Mexico City, Mexico; 2 Division of Clinical Research, “20 de Noviembre” National Medical Centre, ISSSTE, No. 540, Félix Cuevas, Colonia del Valle Sur, Alcaldía Benito Juárez, P.O. 03229, Mexico City, Mexico; 3 Internal Medicine Department, Hospital General de Zona 32. Calzada del Hueso s/n, Coapa, Santa Úrsula Coapa, Alcaldía Coyoacán, P.O. 04980, Mexico City, Mexico; 4 Internal Medicine Department, Tláhuac General Hospital, Mexico City Health Department, No. 655, Avenida la Turba, Villa Centroamericana I, Alcaldía Tláhuac, P.O. 13250, Mexico City, Mexico; 5 National Institute of Medical Sciences and Nutrition “Salvador Zubirán”, National Health Institutes, No. 15, Vasco de Quiroga, Belisario Domínguez Sección 16, Alcaldía Tlalpan, P.O. 14080, Mexico City, Mexico; 6 Internal Medicine Department, Xoco General Hospital. Mexico City Health Department. Avenida México Coyoacán S/N, General Anaya, Alcaldía Benito Juárez, P.O. 03340, Mexico City, Mexico; 7 Specialized Clinic for the Management of the Diabetic Patient “Dr. Manuel González Rivera”. Calle Manuel Carpio y Calle Salvador Díaz Mirón, Plan de San Luis, Santo Tomas, Alcaldía Miguel Hidalgo, P.O. 11340, Mexico City, Mexico

**Keywords:** Urinary transferrin, Chronic kidney disease, Type 2 Diabetes mellitus, Carotid intima-media thickness, Endothelial dysfunction

## Abstract

**Background:**

Albumin, along with other proteins, is abnormally eliminated via the urine during early stages of diabetic nephropathy. Moreover, endothelial dysfunction (ED) accompanying early diabetic nephropathy may develop even before microalbuminuria is detectable. Transferrin has a molecular weight comparable to albumin, whereas transferrinuria and microalbuminuria in a 24-h urine sample may comparably reflect early diabetic nephropathy. Whereas transferrin metabolism is related with ED during very early diabetic nephropathy has not been elucidated yet. This case–control study aimed to evaluate the relation between ED and urine transferrin, even before early diabetic nephropathy is present.

**Methods:**

Patients were enrolled from two study sites in Mexico City: Ticomán General Hospital (healthy controls); and a Specialized Clinic for the Management of the Diabetic Patient (cases). All patients provided written informed consent. The primary endpoint was the correlation between urinary transferrin concentration and ED measured in type 2 diabetic patients without albuminuria. ED was evaluated by ultrasonographic validated measurements, which included carotid intima-media thickness (CIMT) and flow mediated dilation (FMD). Plasma biomarkers included glycated hemoglobin, creatinine, cholesterol and triglycerides, as well as urine albumin, transferrin and evidence of urinary tract infection.

**Results:**

Sixty patients with type 2 Diabetes Mellitus (t2DM; n = 30) or without t2DM (n = 30), both negative for microalbuminuria, were recruited. The group with t2DM were older, with higher values of HbA1c and higher ED. This group also showed significant differences in urine transferrin and urine/plasma transferrin ratio, as compared with healthy controls (14.4 *vs.* 18.7 mg/mL, p = 0.04, and 74.2 *vs.* 49.5; p = 0.01; respectively). Moreover, urine transferrin correlated with higher CIMT values (r = 0.37, p = 0.04), being particularly significant for t2DM population. CIMT also correlated with time from t2DM diagnosis (r = 0.48, p < 0.001) and HbA1c (r = 0.48; p < 0.001).

**Conclusion:**

Urine transferrin correlated with subclinical atherogenesis in patients with t2DM without renal failure, suggesting its potential to identify cardiovascular risk in patients at very early nephropathy stage without microalbuminuria.

## Background

Chronic Kidney Disease (CKD) is a syndrome defined by the persistent alterations in the kidney structure and/or function, with clinical implications. One of its causes is associated with type 2 Diabetes Mellitus (t2DM), and identification of early renal damage may help to evaluate the efficacy of different interventions allowing the delay of diabetic nephropathy in subjects at risk [1].

Microalbuminuria is recognized as a valuable marker of incipient diabetic nephropathy. Early diabetic nephropathy is characterised by a pathological increase in glomerular permeability; then then albumin and other plasma proteins like transferrin, leak from the plasma into the urine. Transferrin’s molecular weight is comparable to that of albumin, but it has a higher isoelectric point [2]. Moreover, Masao et al. found a positive correlation between tubular dysfunction, as evaluated by (1) microglobulin, (2) microglobulin and N-acetyl-D-glycosaminidase, with the degree of microalbuminuria and transferrinuria in patients with diabetic nephropathy [3]. As a matter of fact, the relationship of microalbuminuria and transferrinuria with early tubular damage was further demonstrated quantitatively in biopsy proven diabetic nephropathy [4].

Presence of both microalbuminuria and transferrinuria suggests early dysfunction of glomerular permeability and tubular function, as demonstrated in biopsy proven nephropathy, and correlating with other proteins such as alfa-1 microglobulin, beta-2 microglobulin and N-acetyl-D-glycosaminidase. This also implies a common pathophysiological mechanism underlying transferrin and albumin urine leakage in 24 h sample, once the diabetic renal disease is established; however, there is a lack of information regarding whether the patients without albuminuria would present an early endothelial damage that could be measured with other proteins such as transferrin.

Endothelial dysfunction (ED) and increased cardiovascular risk have been intimately related with early stages of CKD. It has been found that microalbuminuria is associated with ED, although approximately 20% of patients will develop ED and CKD before microalbuminuria is present [5]. There are few studies showing the relationship between microalbuminuria and ED/subclinical atherogenesis, whereas the relation between transferrinuria and ED is less clear and has not been explored in patients with t2DM with very early stages of CKD [6].

One way to explore this relationship between ED and urinary transferrin in t2DM patients is possible using imaging techniques that can be used as a surrogate marker of vascular dysfunction like flow mediated dilation (FMD) and imaging techniques evaluating subclinical atherogenesis as the carotid intima-media thickness (CIMT) measurement [7]. These tests have shown adequate cardiovascular risk stratification, primarily on events such as infarction, use of lipid lowering therapy and assessment of drug efficacy; yet none of them have been evaluated in patients with renal disease, or in diabetic patients at risk of diabetic nephropathy [8].

Thus, we conducted a case–control study to evaluate the relation between transferrinuria with early vascular damage in patients with t2DM before the presence of microalbuminuria and the decline in glomerular filtration rate.

## Methods

### Aim, design and setting

The aim of this study was to evaluate the correlation between vascular damage markers and urine transferrin in patients without diabetic nephropathy. For this purpose, a case–control study was designed and conducted at two sites in Mexico City: Ticomán General Hospital (n = 30, healthy controls); and a Specialized Clinic for the Management of the Diabetic Patient (n = 30, cases).

### Study population

Cases were constituted by patients diagnosed with t2DM, median aged 56.7 years old (IQR 49.2, 64.2), 50% males. On the other hand, controls were constituted by non-diabetic subjects (HbA1c < 6.5%), median aged 42.2 years old (IQR 30.7, 50.5), 33.3% males. Patients were excluded if evidence of nephropathy (increased serum creatinine and/or microalbuminuria), or presence of leucocytes/nitrates in urine sample. All patients provided written informed consent.

### Measurements and endpoints

The primary endpoint was the correlation between transferrinuria and vascular damage measured in patients without albuminuria. ED was evaluated by ultrasonographic validated measurements, which included carotid intima-media thickness (CIMT) and flow mediated dilation (FMD).

All patients were interviewed to meet the clinical inclusion criteria; afterwards, they provided a urinary sample to be tested for urinary tract infection (UTI) for proteinuria. These tests yielded 2 results, patients who tested positive for proteinuria or UTI were excluded; but those who tested negative, were included in the study. The urinary sample was then conserved at 4 °C for subsequent analyses. Then, participants were interviewed and blood sample was collected through venipuncture, and programmed for ultrasonographic evaluation.

Urinary collection was performed from a single urine sample. Briefly, the patients were informed on how to collect the urine and they were given a sterile cup for collection. Participants also received indications regarding sample handling. Patients provided urine sample (approximately volume 20–50 ml) and the investigator, or qualified personnel, processed and interpreted urinalyses results. Urine samples were analyzed as follows: samples were tested by urinary colorimetric dipstick for the presence of nitrates (detection range: negative, traces, positive), proteins (detection range: from negative, traces, up to 1000 mg/dL) and leucocytes (detection range: from negative, traces up to 500 cells/uL).

Regarding blood sample, 10 ml of venous blood was obtained. Complete blood cell count were analyzed through a D×H 900 hematology analyzer (Becker Coulter) using through VCS 360 technology, DataFusion. Plasma chemical analyses (creatinine, glycated hemoglobin, cholesterol, and triglycerides) were analyzed through the D×C 700 AU (Becker Coulter) which uses spectrophotometry principles over a wide wavelength range.

Urine and transferrin determinations were performed by Human Transferrin ELISA Kit ab108911, following providers recommendations.

Ultrasound measurements: For both groups we performed ultrasonographic measurements of carotid internal medial thickness (CIMT) and flow mediated dilation (FMD). These measurements were performed by the investigator with the use of a portable transductor (with wireless connection, WiFi type, 7.5 mHz, for use on cell phone equipment). For CIMT measurements, longitudinal images of the carotid arteries were obtained in which the leading edges of the lumen-intima and media-adventitia interfaces (the “double-line pattern”) of the arterial wall represent intima-media complex, and a part were the measurement was obtained. For FMD (Brachial artery ultrasound) measurements, the test was performed inflating a blood pressure cuff at suprasystolic pressures for five minutes, occluding the upper arm proximal to the ultrasound measurement. Upon the release of the occlusion, an increase in shear stress results in an endothelial-dependent, nitric oxide NO-driven, flow-mediated dilation (FMD) of the brachial artery. The diameter of the artery was assessed before and after occlusion with results being reported as a percent change from baseline.

Statistical analyses were performed using IBM SPSS *Statistics* statistical package v.25.1 as well as GraphPad Prism 8.2.1. Data normality was assessed using Shapiro-Wilks or Kolmogorov–Smirnov tests. Data were resumed as median and interquartile range (IQR) or n (%), for quantitate and categorical variables, respectively. Inferential analyses were performed by independent T-test, Pearson correlation and Fisher’s exact test; using two-tailed *p* value, considering statistical significance p < 0.05.

## Results

A total number of 60 patients with t2DM (n = 30) or without t2DM (n = 30), all negative for CKD, constituted the study population, whose baseline characteristics are shown in Table 1. The group with t2DM were older, with higher values of HbA1c, heterogeneous microvascular complications and higher ED/vascular damage, as assessed by indicators like FMD and CIMT. At the same time, urine transferrin and urine/plasma transferrin of t2DM population showed significant differences (14.4 *vs.* 18.7 mg/mL, p = 0.04, and 74.2 *vs.* 49.5; p = 0.01; respectively).

**Table 1:** Baseline characteristics of the study population

	t2DM (n = 30)	non-t2DM (n = 30)	p-value
Age (y–o)	56.7 (49.2, 64.2)	42.2 (30.7, 50.5)	**0.01**
Age > 65 years	6 (20.0%)	2 (6.7%)	**0.01**
Male	15 (50.0%)	10 (33.3%)	0.1
Weight (kg)	67.8 (54.3, 77.1)	70.5 (58.7, 84.2)	0.59
Height (cm)	158.0 (150.0, 168.2)	161.6 (153.0, 168.5)	0.37
BMI (kg/m^2^)	26.7 (23.7, 28.5)	26.7 (23.3, 29.0)	0.9
Comorbidities			
Hypertension	0 (0)	2 (6.7%)	0.39
Dyslipidemia	11 (36.7%)	16 (53.3%)	0.20
Blood chemistry			
Cholesterol (mg/dL)	175.8 (150.5, 204.7)	175.1 (141.7, 203.7)	0.9
Triglycerides (mg/dL)	146.9 (94.0, 153.0)	147.2 (94.0, 189.0)	0.9
Hb_A1c_ (%)	8.3 (6.4, 9.6)	N. A	**0.01**
Smoking history	13 (43.3%)	10 (33.3%)	0.41
Time from t2DM onset (years)	10.9 (5.0, 15.0)	N. A	**0.01**
T2DM related vascular complications			
None	23 (76.7%)	N. A	**0.01**
Retinopathy	1 (3.3%)		
Neuropathy	2 (6.7%)		
Combined	4 (13.3%)		
T2DM drug therapy			
MET	3 (10.0%)	N. A	**0.01**
Insulin	2 (6.7%)		
Insulin + MET	9 (30.0%)		
Insulin + MET + other*	7 (23.3%)		
MET + other*	7 (23.3%)		
Insulin + other*	2 (6.7%)		
Renal function			
Serum creatinine	0.76 (0.67, 0.83)	0.75 (0.67, 0.81)	0.76
GFR (mL/min)	99.3 (74.6, 120.1)	118.06 (84.75, 146.65)	0.06
Transferrinuria (mg/ml)	14.4 (0.7, 28.8)	18.7 (12.7, 25.3)	**0.04**
Transferrinemia (mg/ml)	2.7 (0.2, 17.0)	2.3 (0.3, 2.2)	0.27
Transferrinuria/transferrinemia Index	74.2 (0.0, 126.4)	49.5 (5.1, 68.7)	**0.01**
Endothelial dysfunction and atherogenesis			
FMD value (%)	9.9 (3.2, 16.0)	19.5 (10.9, 26.2)	**0.01**
CIMT value (mm)	1.19 (0.9, 1.3)	0.66 (0.5, 0.8)	**0.01**
ABI value	1.25 (1.14, 1.35)	1.19 (1.12, 1.26)	0.14

Values shown were tested for parametric distribution. Data is described as number and percentage as follows n (%), as well as median and interquartile range n (q25, q75) depending on the distribution of variables

*BMI* Body mass index, *MET* Metformin, *FMD* Flow mediated dilation measured by ultrasound, *CIMT* Carotid medial thickness measured by ultrasound, *ABI* Ankle brachial index, *T2DM* Type 2 diabetes, *GFR* Glomerular filtration rate measured by Cockcroft-Gault equation

*The drug therapy included under the classification of “other” includes SLGT-2 and DPP-4 Inhibitors. Values highlighted in bold represent statistical significance

**Fig. 1:**
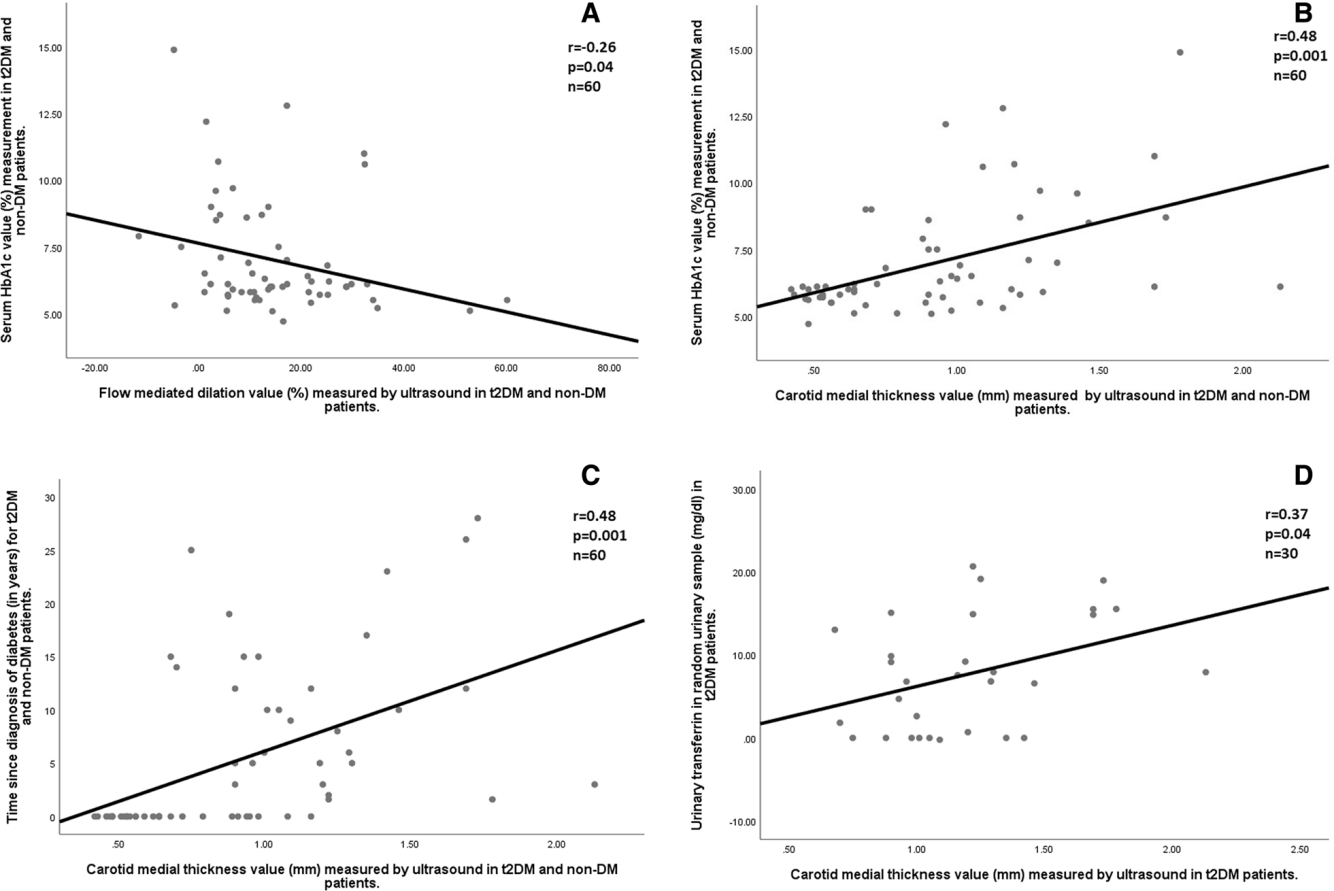
Correlations between ultrasound measurements, serum, and urinary samples. **A** shows the correlation between serum HbA1c and FMD values in the total study group. **B** shows the correlation between serum HbA1c values and CIMT values in the total study group. **C** shows the correlation between time since diagnosis of diabetes an CIMT value in the total study group. **D** shows the correlation between transferrinuria in random urine sample in t2DM patients and CIMT values in t2DM patients. At the top right corner of each panel, it is shown the correlation factor (r) statistical significance (p-value), and the total of patients evaluated for each correlation (n)

After several correlation analyses, urine transferrin was found to correlate with higher CIMT values (r = 0.37, p = 0.04), being particularly significant for t2DM population (Fig. 1D). Likewise, time from t2DM diagnosis also correlated with CIMT (r = 0.48, p < 0.001, Fig. 1C); while HbA1c correlated with both CIMT and FMD (r = 0.48; p < 0.001, and r = − 0.26, p = 0.04; respectively, Fig. 1A and B).

During exploration of factors associated with ED/vascular damage, subclinical atherogenesis was related with male sex with differences according to t2DM, as well as dyslipidemia, time from t2DM diagnosis and HbA1c. Likewise, ED/vascular damage was linked to time from t2DM diagnosis (Table 2). Urine transferrin did not show significant association during this exploring method.

**Table 2:** Risk factors associated with endothelial damage

	CIMT > 0.7 mm			FMD < 11%		
	n (%)	OR (CI 95)	p-value	n (%)	OR (CI 95)	p-value
Age > 65						
All	6 (10%)	1.2 (0.2–5.7)	0.73	5 (8.3%)	1.7 (0.4–7.4)	0.42
Type 2 DM	6 (20%)	0.2 (0.01–5.03)	0.38	4 (13.3%)	0.7 (0.12–3.99)	0.69
Non-DM	0	N. A	–	1 (3.3%)	3.6 (0.19–67.6)	0.38
Sex male						
All	20 (33.3)	4.2 (1.2–13.8)	**0.01**	12 (20%)	1.3 (0.4–3.9)	0.53
Type 2 DM	14 (46.6%)	1 (0.05–17.6)	1.00	10 (33.3%)	1.3 (0.3–5.91)	0.7
Non-DM	6 (20%)	8.5 (1.4–49.5)	**0.01**	2 (6.6%)	0.7 (0.11–4.7)	0.7
Smoking						
All	14 (23.3%)	0.9 (0.3–2.7)	0.9	13 (21.6%)	2.4 (0.8–6.9)	0.11
Type 2 DM	11 (36.6%)	N. A	–	10 (33.3%)	2.9 (0.59–14.72)	0.18
Non-DM	3 (10%)	1 (0.19–5.2)	1	3 (10%)	1.7 (0.3–9.7)	0.54
Hypertension						
All	2 (3.3%)	N. A. (0-INF)	–	1 (1.6%)	1.3 (0.07–22.1)	0.84
Type 2 DM	0	N. A	–	0	N.A	–
Non-DM	2 (6.6%)	N. A. (0-INF)	–	1 (3.3%)	3.6 (0.19–67.6)	0.38
Dyslipidemia (F3)						
All	6 (10%)	0.69 (0.18–2.6)	0.59	3 (5%)	0.4 (0.1–1.7)	0.24
Type 2 DM	4 (13.3%)	N. A. (0-INF)	–	2 (6.6%)	0.5 (0.06–4.4)	0.55
Non-DM	2 (6.6%)	0.9 (0.14–5.9)	0.90	1 (3.3%)	0.4 (0.04–4.7)	0.52
Dyslipidemia (F4)						
All	2 (3.3%)	0.16 (0.03–0.88)	**0.03**	4 (6.6%)	1.3 (0.3–6.05)	0.68
Type 2 DM	2 (6.6%)	0.07 (0.003–1.7)	0.1	2 (6.6%)	1.1 (0.09–14.6)	0.90
Non-DM	0	N. A	–	2 (6.6%)	2.6 (0.3–20.5)	0.34
BMI > 25						
All	25 (41.6%)	1.3 (0.4–3.9)	0.59	18 (30%)	1.3 (0.4–4.1)	0.54
Type 2 DM	18 (60%)	1.8 (0.1–31.9)	0.68	13 (43.3%)	1.8 (0.3–8.3)	0.44
Non-DM	7 (23.3%)	2.1 (0.3–13.04)	0.40	5 (16.6%)	1.3 (0.2–8.4)	0.76
Time from DM						
All	28 (46.6%)	32.6 (6.3–167.2)	**0.001**	19 (31.6%)	5.6 (1.8–17.4)	**0.01**
Type 2DM (> 5 years)	22 (73.3%)	N. A	–	14 (46.6%)	0.2 (0.02–2.7)	0.27
Non-DM	0	N. A	–	0	N. A	–
Hb_A1c_ > 8.3%						
All	12 (20%)	5.04 (1.01–25.09)	**0.04**	9 (15%)	3.07 (0.8–10.6)	0.07
Type 2 DM	12 (40%)	N. A	–	9 (30%)	1.08 (0.2–4.7)	0.90
Non-DM	0	N. A	–	0	N. A	–
Transferrinuria						
All	16 (26.6%)	0.69 (0.24–1.98)	0.50	11 (18.3%)	0.73 (0.26–2.05)	0.55
Type 2 DM	13 (43.3%)	0.86 (0.04–15.2)	0.92	9 (30%)	1.08 (0.24–4.7)	0.90
Non- DM	3 (10%)	0.45 (0.08–2.3)	0.34	2 (6.6%)	0.36 (0.05–2.2)	0.28
Transferrinemia						
All	5 (8.3%)	0.74 (0.17–3.1)	0.68	2 (3.3%)	0.32 (0.06–1.6)	0.18
T2DM	3 (10%)	N. A	–	2 (6.6%)	1.1 (0.09–14.6)	0.9
Non-DM	2 (6.6%)	1.2 (0.17–8.2)	0.84	0	N. A	–

Data is described as number and percentage as follows n (%)

*OR* Odds ratio, *CI* Confidence interval, *BMI* Body mass index, *F3* Frederickson type 3, *F4* Frederickson type 4

Values highlighted in bold represent statistical significance

*For the odds ratio calculated in the overall group, the values were taken from the groups as having diabetes or being non-diabetic with the values equals to 0 for the non-diabetic group. For the odds ratio calculated in the group of type 2 DM, the risk was calculated whether they had more than 5 years of being diagnosed with diabetes

## Discussion

The key finding of the present study is that urinary transferrin was significantly related with subclinical atherogenesis, particularly in patients with diagnosis of t2DM, who had not developed renal disease yet. This was primarily evidenced by a positive correlation between urinary transferrin and CIMT, being particularly significant for subjects with t2DM. Such correlation results in a potentially useful tool for clinical assessment of ED/vascular damage in patients with very early diabetic nephropathy.

Our primary endpoint was to explore whether transferrinuria was related with early ED, known to occur during renal hyperfiltration phase and just before clinically significant events develop, like microalbuminuria and the decline of glomerular filtration rate.

Microalbuminuria has been traditionally used for the assessment of early renal damage in patients with t2DM. However, approximately 30% to 45% of t2DM patients may show a diminished GFR (glomerular filtration rate), even in the absence of albuminuria. Therefore, clinical characterization of new biomarkers is required [9]. To date, the role of transferrinuria as a marker of ED/vascular damage in patients with t2DM without known renal disease has not been described.

Urinary transferrin, as well as several urine biomarkers such as ceruloplasmin, immunoglobulin G, podocalyxin, neutrophil gelatinase-associated lipocalin (NGAL), N-acetyl-beta-glycosaminidase, α-1-microglobulin, 8-hydroxy-deoxyguanosine, tumor necrosis factor-alpha (TNF-α), interleukin-18 and cystatin C, have shown to reflect early renal damage progression when assessed altogether with microalbuminuria [10]. However, urinary transferrin alone may also correlate with early vascular damage reflected by ED and subclinical atherogenesis, leading to arterioles inability of adaptive vasodilation and to progressive increase of vascular stiffness [11]. This indicates that urinary transferrin is an early marker of vascular disease, occurring even before overt microalbuminuria in patients with t2DM.

In the present study, patients showed an apparently “normal” GFR without microalbuminuria. The relation between GFR and microalbuminuria, during very early phases of diabetic renal damage is not yet clear. Previous reports describe that early decline of renal filtration rate may occur in the absence of microalbuminuria in approximately 30% to 45% of cased with t2DM, which may be associated to an early renal hyperfiltration rate, denoting renal ED.

Consistently, a concept of a “renal reserve” has been proposed, which describes that a reserved renal function can be utilized when the physiological demand for single nephron GFR increases. This concept also supports that in subclinical renal disease like t2DM, at stage before basal GFR begins to reduce, renal functional reserve can be recruited in a manner that preserves renal function. The extension of this concept is that once a decline in basal GFR can be detected, renal disease is already well progressed [12]. We acknowledge that we did not perform a direct measure of renal ED, such as determination of vasoactive mediators, either in plasma or urine. However, studies of patients on hemodialysis, showing that regression of CIMT occurs after renal transplant [13], as well as the role of carotid plaques as predictors of renal outcomes in individuals with t2DM without other microvascular complications [14] strongly suggest the relation between ED and accelerated subclinical atherogenesis, as assessed by CIMT.

While the relation of urinary transferrin with early renal damage and subclinical atherogenesis is not completely clear, possible explanation is acting through different pathological mechanisms. Although the underlying problem often cannot be treated, extensive experimental models and human studies suggest that progressive CKD may be largely due to secondary factors that are sometimes unrelated to the activity of the initial disease [15]. Some of these secondary factors include: (a) compensatory response to nephron loss by maintaining total GFR; (b) direct endothelial cell damage, like that induced by systemic hypertension; and c marked tubulointerstitial injury (tubular dilatation, interstitial fibrosis), even if the primary process is a glomerulopathy [16].

Zylka et al*.* found the highest relationship between biomarkers associated with glomerular damage (in comparison to albuminuria) and diminished GFR, evaluated in a population similar to our study. Such biomarkers included transferrin, immunoglobulin G, ceruloplasmin, type IV collagen, glycosaminoglycans, prostaglandin D synthase lipocalin type, fibronectin, vascular endothelial growth factor, cystatin C, and nefinina [17]. Besides, Kim et al. described a correlation between tubulointerstitial damage in t2DM with nephropathy, including different biomarkers such as urinary transferrin, that could lead to evaluate histopathological damage [18]. Therefore, the correlation of urinary transferrin with ED suggests that glomerular and tubulointerstitial damages are related with vascular dysfunction, and urinary transferrin may be a useful marker of such dysfunction.

A specific study is required to determine whether urine leakage of transferrin and albumin may occur in the absence of endothelial damage. Unfortunately, the present study design did not allow exploring such specific mechanism of damage.

In addition, our results suggest that either the urinary transferrin or the urinary/index transferrin ratio are useful to identify patients with diabetic nephropathy at early risk for ED/vascular damage. These findings suggess the dynamic participation of the whole transferrin metabolism as related with early renal and vascular damages. The clinical usefulness of urine transferrin and plasma/urine transferrin ratio in the evaluation of early renal damage and subclinical atherogenesis, as a part of a routine test to identify high risk population with t2DM, are to be developed, and deserves further evaluation.

Interestingly, ED was more associated with several t2DM-related variables than urine transferrin during exploratory risk factor analyses. Since this analysis requires that variables are dichotomic and considering the significant correlation between urine transferrin and CIMT, it is suggested that optimal cutoff is still to be established, which at the present study design was not able to address.

The main limitation of our study was the heterogeneous characteristics between cases and controls within the study population, particularly in terms of age. However, no significant association was found between age and FMD nor CIMT values that could affect the study results. Another limitation was the determination of albuminuria with a bedside urine dipstick test in a urine single sample. Since this method is more sensitive for albumin (even with the false positive, false negative results that can occur), it resulted adequate for our analysis. Finally, we used urine transferrin measured in a single morning urine sample, which did not show significant difference with 24 h urine transferrin.

In conclusion, urine transferrin may be related with early ED and subclinical atherogenesis in patients with t2DM at a very early renal damage. This relation may be useful to stratify patients at higher vascular risk as well as early nephropathy, even before microalbuminuria becomes evident.

## Data Availability

The datasets generated and analyzed during the current study are not publicly available due to privacy policies of the hospital and patient’s information; but are available from the corresponding author on reasonable request.

## Abbreviations

CKD: Chronic kidney disease
ESRD: End stage renal disease
QOL: Quality of life
ED: Endothelial dysfunction
AKI: Acute kidney injury
GFR: Glomerular filtration rate
CKD-EPI: Chronic kidney disease epidemiology collaboration
CIMT: Carotid intima media thickness
FMD: Flow mediated dilation
T2DM: Type 2 Diabetes mellitus
HbA1c: Glycated hemoglobin
BMI: Body mass index
MET: Metformin
GFR: Glomerular filtration rate
ABI: Ankle brachial index
OR: Odds ratio
CI: Confidence interval
DM: Diabetes mellitus
NA: Not applicable
